# Clinical and therapeutical significances of the cluster and signature based on oxidative stress for osteosarcoma

**DOI:** 10.18632/aging.205354

**Published:** 2023-12-29

**Authors:** Mengjie Ding, Xianting Ran, Siyu Qian, Yue Zhang, Zeyuan Wang, Meng Dong, Zhenzhen Yang, Shaoxuan Wu, Xiaoyan Feng, Jieming Zhang, Linan Zhu, Songtao Niu, Xudong Zhang

**Affiliations:** 1 Department of Oncology, The First Affiliated Hospital of Zhengzhou University, Zhengzhou, Henan, China; 2 Department of Endocrinology and Metabolism, The First Affiliated Hospital of Zhengzhou University, Zhengzhou, Henan, China

**Keywords:** osteosarcoma, oxidative stress, bioinformatics, prognosis, immune

## Abstract

Background: It is of great clinical significance to find out the ideal tumor biomarkers and therapeutic targets to improve the prognosis of patients with osteosarcoma (OS). Oxidative stress (OXS) can directly target intracellular macromolecules and exhibit dual effects of tumor promotion and suppression.

Methods: OXS-related genes (OXRGs) were extracted from public databases, including TARGET and GEO. Univariate Cox regression analysis, Random Survival Forest algorithm, and LASSO regression were performed to identify prognostic genes and establish the OXS-signature. The efficacy of the OXS-signature was further evaluated by Kaplan-Meier curves and timeROC package. Evaluation of immunological characteristics was achieved based on ESTIMATE algorithm and ssGSEA. Submap algorithm was used to explore the response to anti-PD1 and anti-CTLA4 therapy for OS. Drug response prediction was conducted by using pRRophetic package. The expression values of related genes in the OXS-signature were detected with PCR assays.

Results: Two OXS-clusters were identified for OS, with remarkable differences of clusters presented in prognosis. Kyoto Encyclopedia of Genes Genomes (KEGG) analysis showed that differentially expressed genes (DEGs) between the OXS-clusters were significantly enriched in several immune-related pathways. Patients with lower OS-scores attained better clinical outcomes, and presented more sensitivity to ICB therapy. By contrast, OS patients with higher OS-scores revealed more sensitivity to certain drugs. Furthermore, critical genes, RHBDL2 and CGREF1 from the model, were significantly higher expressed in OS cell lines.

Conclusions: Our study identified the clusters and signature based on OXS, which would lay the foundation for molecular experimental research, disease prevention and treatment of OS.

## INTRODUCTION

Osteosarcoma (OS) develops from the mesenchymal cell line and is the most common primary bone malignancy in adolescents [1]. The main clinical manifestations of OS contain fever, discomfort, weight loss, anemia and organ failure [1]. Generally, OS is characterized with low survival rate, poor general condition, and non-ideal treatment effects [1, 2]. The growth rate of individual patients with OS is very fast, which comes with the possible occurrence of early lung metastasis, eventually leading to systemic deterioration [3]. Even after amputation and chemotherapy, about 40% of OS patients still die of lung metastasis [4]. According to the WHO classification of bone neoplasms in 2020, the histological classification of OS includes conventional osteosarcoma, telangiectatic osteosarcoma, small cell osteosarcoma, parosteal osteosarcoma, periosteal osteosarcoma, high-grade surface osteosarcoma, and secondary osteosarcoma [5]. Despite the development of new treatment methods for OS with the advancement of medical technology, the five-year survival rate of clinical patients has not improved significantly [6]. Therefore, it is of great clinical significance to figure out the ideal tumor biomarkers and therapeutic targets to improve the prognosis and prolong the survival of patients with OS.

Bioinformatics analysis is a discipline that can store, classify, refine and analyze biological information in large databases with the aid of computers in scientific research in the field of biology. As an interdisciplinary subject between biomedical field and computer field, it is one of the frontier fields at present, and also the core field of life science in the 21st century. Bioinformatics analysis focuses on genomics and proteomics, which aims to analyze the biological information related to gene expression as well as protein structure and function based on nucleic acid and amino acid sequences. With the rapid development of gene chip technology to further promote the fast progress of the bioinformatics technique, through relying on large databases, it has provided the advantageous support for life science data research, realized data collection, screening, editing and management through research tools (computer), and facilitated calculation and analysis of corresponding results, and visualization. Bioinformatics analysis can contribute to analyzing and identifying key target genes more conveniently, accurately and intuitively in massive gene data, and predicting the impact of important genes on signaling pathways or target molecules in biological behaviors. In the field of cancer research, the Gene Expression Omnibus (GEO) database, Surveillance, Epidemiology, and End Results (SEER) database, the cancer genome atlas (TCGA) database and the Database for Annotation, Visualization and Integrated Discovery (DAVID) database have been established. Whether it is the differential expression prediction of tumor-related genes, the enrichment of key signaling pathway molecules, or the survival analysis of clinical relevance, the importance and potential of bioinformatics for cancer research become evident.

Oxidative stress (OXS) is a common stress state in tumors, which refers to the imbalance between oxidation and anti-oxidation in the body, and the tendency to oxidation, thus resulting in abnormal oxidative signal regulation mechanism and macromolecular oxidative damage [7]. The increase of reactive oxygen species (ROS) level is the primary factor leading to cellular OXS [8]. OXS is the main culprit of cell damage, which can directly target intracellular macromolecules and demonstrate dual effects of tumor promotion and tumor suppression [7, 9–11]. The regulation of redox homeostasis of tumor cells can significantly affect the therapeutic effect of tumor, which is considered as a potential clinical treatment strategy. However, the regulation of tumor cell fate by OXS is highly complex and closely related to tumor types and tumor pathogenesis. This suggests how to accurately control the anti-tumor effect of OXS and avoid the tumor-promoting effect is still the key to subsequent research. With the continuous application of bioinformatics and other disciplines related to big data analysis, it is expected to further analyze the heterogeneity of OXS in tumors, and then discover and investigate more therapeutic targets.

In this study, based on multiple bioinformatics databases, the clusters and signature based on OXS were established using data from Therapeutically Applicable Research to Generate Effective Treatments (TARGET), OXS-related genes (OXRGs), Gene Ontology (GO) enrichment analysis, Kyoto Encyclopedia of Genes and Genomes (KEGG) enrichment analysis, survival analysis, Least Absolute Shrinkage and Selection Operator (LASSO) analysis. Furthermore, the prognosis, treatment strategy and immunological characteristics of the OXS-signature were also explored. The expression values of related genes in the OXS-signature were detected by PCR assays. Our study would lay the foundation for molecular experimental research, disease prevention and treatment of OS.

## MATERIALS AND METHODS

### Data collection and preprocessing for osteosarcoma

Since it was not possible to combine gene expression and clinical prognosis analysis due to the lack of clinical data of OS in TCGA database, RNA expression information and clinical data involved in OS were obtained from TARGET (https://ocg.cancer.gov/programs/target) [12], containing a total of 85 samples with OS. The data forms of fragments per kilobase of transcript per million fragments mapped (FPKM) were transformed to transcripts per kilobase million (TPM) [13]. In the meantime, to validate the analysis results, necessary data were also included from the GEO (https://www.ncbi.nlm.nih.gov/geo/). GeneCards (https://www.genecards.org) is a comprehensive database of human genes, collating data from more than 100 websites to provide concise information on all known and predicted human genes in genome, proteome, transcription, genetics, and function [14, 15]. Finally, a total of 80 OXRGs were extracted by setting the threshold of relevance score to be greater than 20 [16]. The processed data was shown in Supplementary File 1.

### Establishing the OXS-clusters and OXS-signature

Univariate Cox regression analysis was conducted on the 80 OXRGs (Supplementary Table 1) extracted to acquire prognostic OXRGs (P<0.05) [17]. Subsequently, clustering analysis was performed on the prognostic OXRGs obtained utilizing ConsensusClusterPlus package in R to identify OXS-related patterns [18].

Differentially expressed genes (DEGs) between OXS-clusters were screened out via limma package (V3.57.11) in R (logFC>1, FDR<0.01) [19] and Univariate Cox regression analysis was carried out on filtrated DEGs to search prognostic DEGs preliminarily (P<0.01) [17]. In order to filtrate DEGs with more valuable prognosis, Random Survival Forest model was necessarily constructed through randomForestSRC (V3.2.2) package in R (variable importance>0.3) [20, 21]. Based on LASSO analysis [22], the regression coefficients of the above genes were estimated and weighted, and OS-score was calculated for each OS sample by combining the expression values of the genes, thus establishing the OXS-signature. Further, the model was created using genes and coefficients corresponding to lambda.min values.

### Evaluating the efficacy of the OXS-signature

After computing the OS-score of each OS, OS patients were distinguished into two subgroups (OS patients with higher OS-scores and OS patients with lower OS-scores) according to the best optimal cutoff. Using Kaplan-Meier curves, differences in prognosis between the two subgroups were assessed by survminer (V0.4.9) package in R, which was verified by an OS cohort from GEO database (GSE21257). To further investigate the efficiency and accuracy of the OXS-signature for prognostic prediction, timeROC (V0.4) package was employed to plot the 1-, 2-, 3-, 4-, and 5-year survival receiver operating characteristic (ROC) curves.

### Analysis of immunological characteristics of OXS-signature

The ESTIMATE score, immune score, and stromal score of each OS patient were calculated by the ESTIMATE algorithm (The Estimation of Stromal and Immune cells in Malignant Tumor tissues using Expression) [23] and the differences between the two subgroups were then compared. The enrichment scores computed by single sample gene set enrichment analysis (ssGSEA) from Gene Set Variation Analysis (GSVA) (GSVA, V1.49.8) package in R [24, 25] were utilized to reflect the infiltration level of 28 immune cells. Submap algorithm was used to explore the response to anti-PD1 and anti-CTLA4 therapy for OS [26–28].

### Enrichment analysis

For human genes, numerous gene sets have been constructed from the perspectives of position, function, metabolic pathway, target combination and so on. A gene set consists of many genes with similar positions or similar functions. MSigDB database stores a large number of gene sets [29]. The gene sets used for KEGG and GO analyses were downloaded from MSigDB [29]. Over Representation Analysis (ORA) and Gene Set Enrichment Analysis (GSEA) analyses were implemented by clusterProfiler package [30].

### Response prediction for chemotherapeutic agents

Data of chemotherapeutic agents used in our study were acquired from the Genomics of Drug Sensitivity in Cancer (GDSC, https://www.cancerrxgene.org/) [31]. The half maximal inhibitory concentration (IC50) values of these chemotherapeutic agents were computed by pRRophetic (V0.5) package in order to assess the response prediction [32].

### Cell culture

The hFOB1.19 cell line was purchased from the American Type Culture Collection (ATCC; Manassas, VA, USA) and was cultured in DMEM/F12 + 0.3mg/ml G418 + 10% FBS (fetal bovine serum) + 1% P/S (penicillin-streptomycin). The OS cell lines U-2 and Saos-2 were purchased from National Collection of Authenticated Cell Cultures (Shanghai, China). Specifically, U-2 was cultured in 90% McCoy’s 5A medium supplemented with 10% FBS while Saos-2 was cultured in 85% McCoy’s 5A medium supplemented with 15% FBS. The mediums needed to be replaced every other day.

### Quantitative real-time PCR

Total RNA from the cell lines was extracted applying RNAsimple Total RNA Kit (Tiangen, Beijing, China). Following RNA extraction, total RNA was reversely transcribed using PrimeScript RT reagent Kit (Takara, Otsu, Japan) to obtain cDNA. Using 2 μL cDNA with SYBR Premix Ex Taq (Takara, Otsu, Japan) and primers, target gene expression was determined by the Applied Biosystems StepOne Plus Real-Time PCR system (Life Technologies, Grand Island, NY, USA). The primers of the target gene were purchased from Sangon Biotech (Shanghai, China). Table 1 lists the sequences.

**Table 1:** The primer sequences in PCR analysis.

**Symbol**	**Sequences (5’-3’)**
RHBDL2-F	TCAGCACATCTTGGGGAATCT
RHBDL2-R	CACTCCTGCCAGGTACACC
CGREF1-F	ACGATGACAGTGTTAATCCTGC
CGREF1-R	CCTAGTCCCTTTAGGTAGCTCTG
C2-F	TGGAAAGTCCAATATGGGTGGC
C2-R	CTGGTGCAGAGCCTTTGTGT
GAPDH-F	GGAGCGAGATCCCTCCAAAAT
GAPDH-R	GGCTGTTGTCATACTTCTCATGG

### Western blot assay

Osteosarcoma cell lines Saos2, U2OS and normal osteoblast cell line hFob1.19 cells were washed with PBS and lysed for 30 min in radioimmunoprecipitation assay buffer (Beyotime Biotechnology, Shanghai, China). Cells were subsequently centrifuged at 12,000 × g for 30 min at 4° C, to collect the supernatant. The protein concentration was measured using the bicinchoninic acid method. Approximately 30 μg of protein was taken and separated by 10% sodium dodecyl sulfate-polyacrylamide gel electrophoresis and further transferred to nitrocellulose filter membranes (General Electric, Chicago, IL, USA). The primary antibodies (RHBDL2 (1:1000, ab236185, Abcam, UK) and β-actin (1:1500, ab8226, Abcam)) purchased from the Abcam were used in the assay. Notably, three independent repeated assays were performed.

### Immunofluorescence

Cells (2.5 × 10^5^) in good condition were fixed with 4% paraformaldehyde at 25° C for 30 min, followed by PBS washing. Afterwards, cells were permeated using 0.3% Triton X-100 at 25° C for 10 min, and then sealed with 3% bovine serum albumin after being washed at 37° C for 30 min. Further, cells were incubated with the primary antibodies RHBDL2 (1:200, ab236185, Abcam) at 37° C for 1 h. After washing, secondary antibodies were added for 1-hour incubation at 37° C. Finally, DAPI was adopted for cell counterstaining, and the images were observed using a fluorescence microscope (Olympus) at 100 × 10 magnification.

### Statistical analysis

Non-normal and normal distribution data should be compared by Wilcoxon and T test, respectively. All heatmaps in this study were drawn by pheatmap (V1.0.12) package in R and ggplot2 (V4.1.2) package in R was employed for visualization. The statistical methods used in the other analyses were mentioned in the corresponding sections above. P<0.05 was considered statistically significant. The code script was shown in Supplementary File 2.

### Data availability

The RNA sequencing profiles are able to be gained from The Cancer Genome Atlas (TCGA) (https://toil.xenahubs.net). Further inquiries can be directed to the corresponding author.

## RESULTS

### Characteristics of OXS-clusters in TARGET-OS

**Figure 1:**
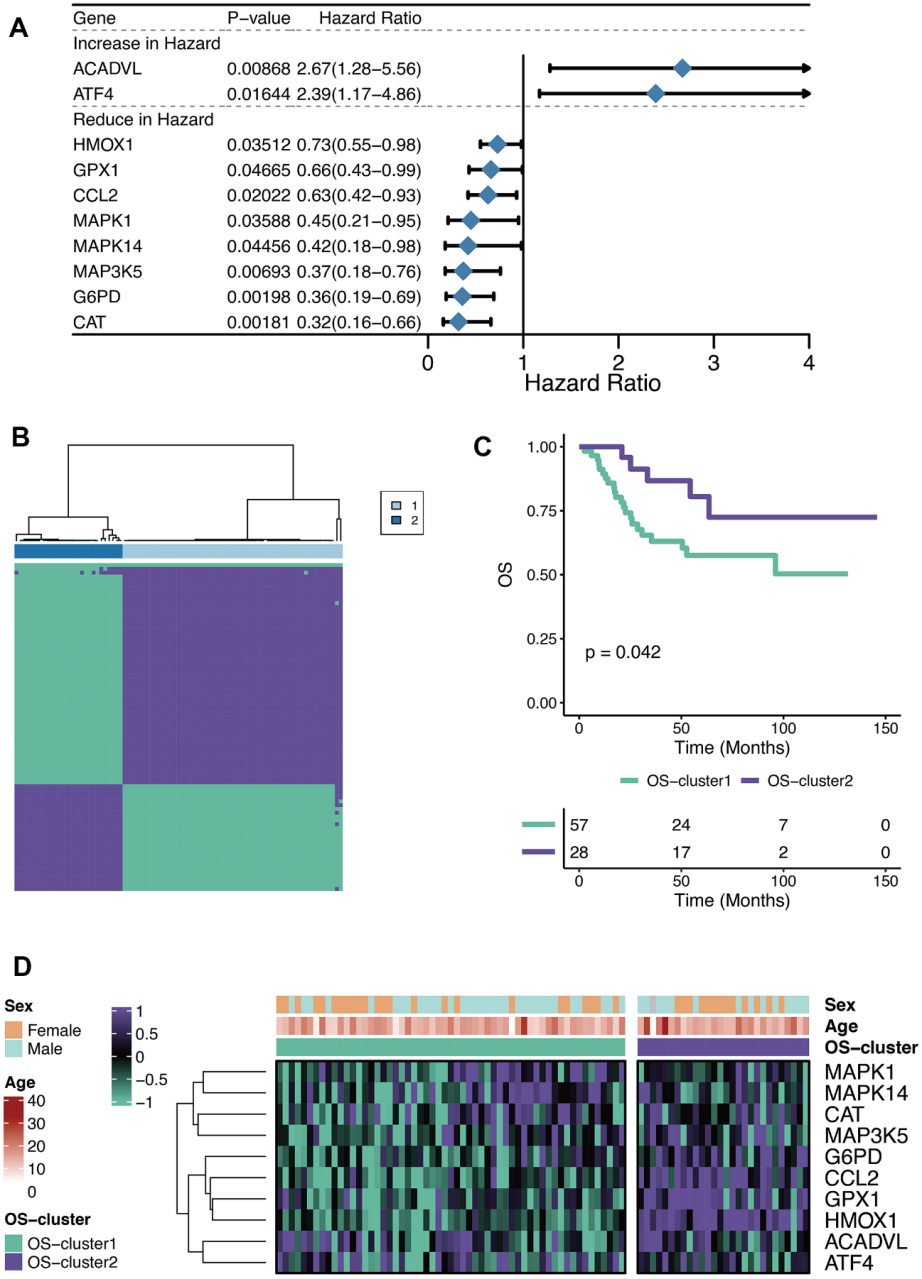
**Characteristics of OXS-clusters in TARGET-OS.** (**A**) Forest plot for Univariate Cox regression analysis of ten prognostic OXRGs; (**B**) Cluster diagram for subtype analysis of osteosarcoma samples, with the intragroup correlations being the highest and the inter-group correlations being low when k=2; (**C**) Kaplan-Meier curve of the correlation between OXS-clusters and survival status of osteosarcoma patients; The green curve denotes OS-cluster1, and the purple curve denotes OS-cluster2. (**D**) The heatmap displaying the distribution of the ten prognostic OXRGs, clinicopathological characteristics (Sex, Age) and OXS-clusters; Purple represents high gene expression, while green represents low gene expression.

The Univariate Cox regression analysis was performed on the 80 OXRGs filtrated by the threshold value (relevance score>20) aforementioned, thus identifying ten prognostic OXRGs (Figure 1A), encompassing two potential oncogenes (HR>1; ACADVL, and ATF4) and eight potential suppressor genes (HR<1; HMOX1, GPX1, CCL2, MAPK1, MAPK14, MAP3K5, G6PD, and CAT). With clustering analysis completed based on ConsensusClusterPlus, the optimal number of clusters (k=2) was determined and two OXS-clusters were generated (OS-cluster1 and OS-cluster2) for subsequent research (Figure 1B). Patients with OS in OS-cluster2 exhibited significantly better clinical outcomes than those in OS-cluster1 (Figure 1C). Compared with OS-cluster1, the expression levels of G6PD, CCL2, GPX1, and HMOX1 became relatively higher in OS-cluster2 (Figure 1D).

### Enrichment analysis of OXS-clusters in TARGET-OS

**Figure 2:**
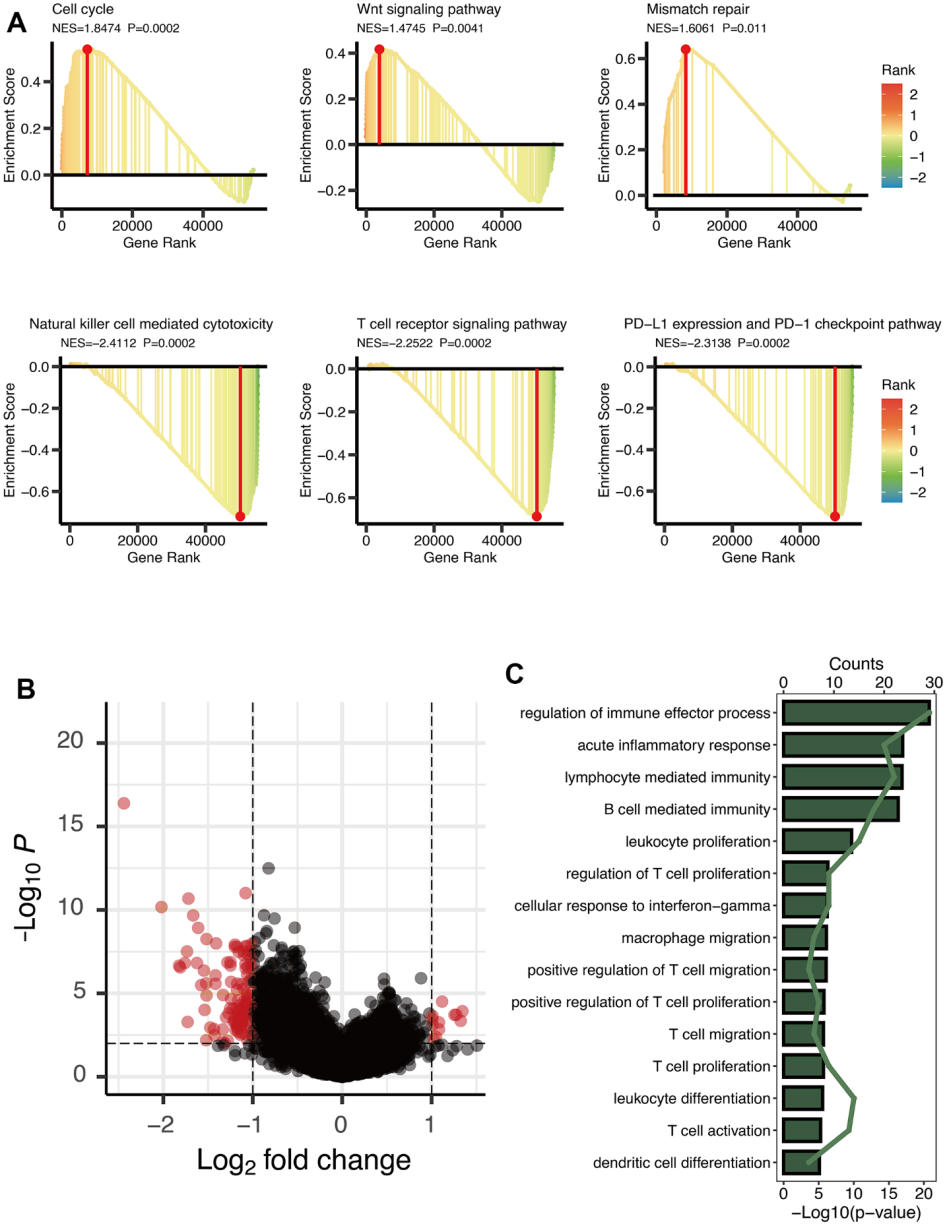
**Enrichment analysis of OXS-clusters in TARGET-OS.** (**A**) GSEA maps of cancer and immune-related signaling pathways for OXS-clusters; (**B**) The volcano map reflecting the differentially expressed genes identified between the two OXS-clusters (logFC>1, FDR<0.01), with the black dots representing genes that do not reach the threshold, and the red dots representing genes that reach the threshold; (**C**) KEGG enrichment map of 125 differentially expressed genes.

GSEA analysis manifested three cancer-related pathways (Cell cycle, Wnt signaling pathway, and Mismatch repair) and three immune-related pathways (Natural killer cell mediated cytotoxicity, T cell receptor signaling pathway, and PD-L1 expression and PD-1 checkpoint pathway) that might be regulated by OXS-clusters, providing insights for exploring the mechanism of the clustering system for OS (Figure 2A). A total of 125 DEGs were observed between the two OXS-clusters and the volcano map was drawn to directly reflect the expression differences of DEGs (Figure 2B). Furthermore, KEGG analysis of the above identified DEGs revealed that these DEGs were significantly enriched in several immune-related pathways, including regulation of immune effector process, acute inflammatory response, lymphocyte mediated immunity, B cell-mediated immunity, leukocyte proliferation, regulation of T cell proliferation, cellular response to interferon-gamma, macrophage migration, positive regulation of T cell migration, positive regulation of T cell proliferation, T cell migration and proliferation, leukocyte differentiation, T cell activation, and dendritic cell differentiation (Figure 2C). In conclusion, OXS-clusters might be closely related to the immune activity of OS.

### Establishment of OXS-signature for TARGET-OS

**Figure 3:**
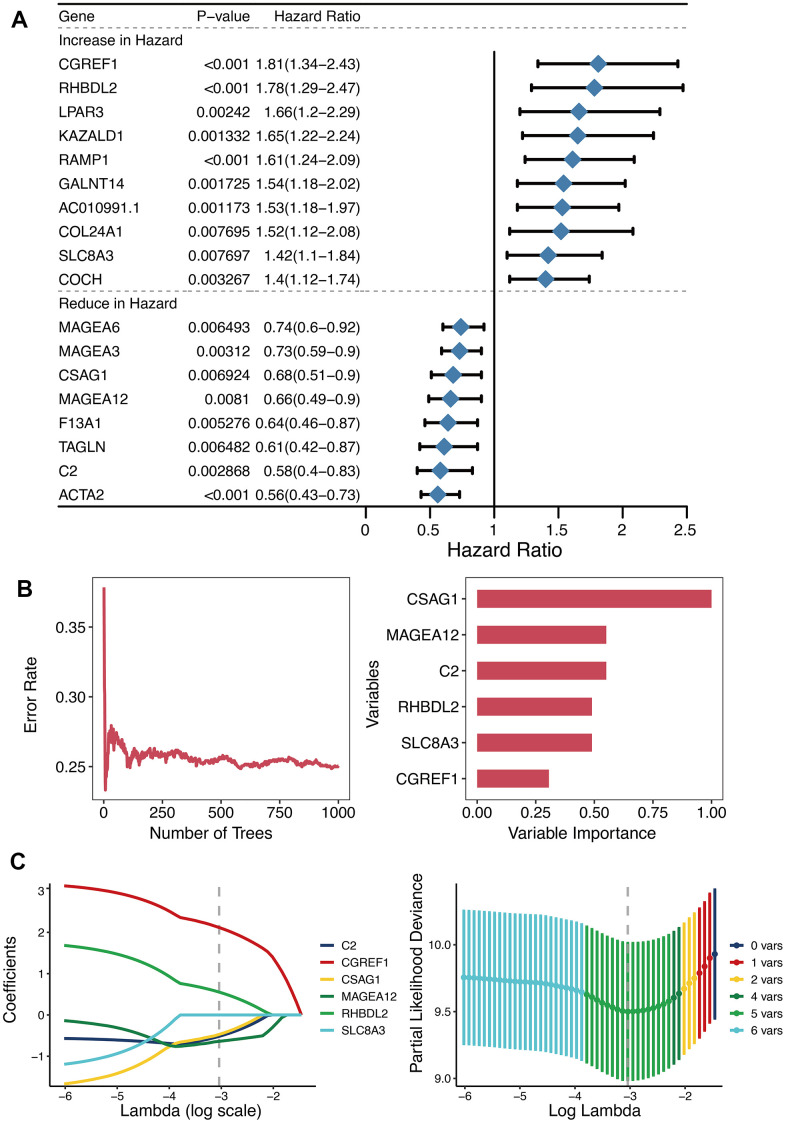
**Establishment of OXS-signature for TARGET-OS.** (**A**) Forest plot for Univariate Cox regression analysis of 18 prognostic genes 125 differentially expressed genes between the two OXS-clusters; (**B**) The distribution of error rates in Random Survival Forest model and the variable relative importance of the six genes. Notably, only genes with variable importance greater than 0.3 were screened out for display. (**C**) Lambda selection diagram for Least Absolute Shrinkage and Selection Operator (LASSO) analysis, with different colored curves representing different genes.

Univariate Cox regression analysis of 125 DEGs between the two OXS-clusters yielded 18 prognostic genes (Figure 3A). Among them, there were ten genes increasing in hazard (HR>1; CGREF1, RHBDL2, LPAR3, KAZALD1, RAMP1, GALNT14, AC010991.1, COL24A1, SLC8A3, and COCH) and eight genes reducing in hazard (HR<1; MAGEA6, MAGEA3, CSAG1, MAGEA12, F13A1, TAGLN, C2, and ACTA2). Hence, ten oncogenic genes and eight tumor-suppressor genes were observed. Whereafter, the distribution of error rates generated by Random Survival Forest model is described in Figure 3B. Based on threshold value (variable importance>0.3), six genes (CSAG1, MAGEA12, C2, RHBDL2, SLC8A3, and CGREF1) with more prognostic potential were screened out (Figure 3B). LASSO analysis was further performed on these six genes to establish the OXS-signature based on the OS-score computed by the following formula: OS-score=–0.4719×CSAG1–0.5174×C2– 0.6393×MAGEA12+0.5535×RHBDL2+2.1171×CGREF1. Therefore, each OS sample was given a corresponding OS-score for subsequent research. The lambda selection diagram is displayed in Figure 3C. The OXS-signature ended up containing five genes: RHBDL2, CGREF1, C2, CSAG1, and MAGEA12.

### Efficacy of OXS-signature for OS

**Figure 4:**
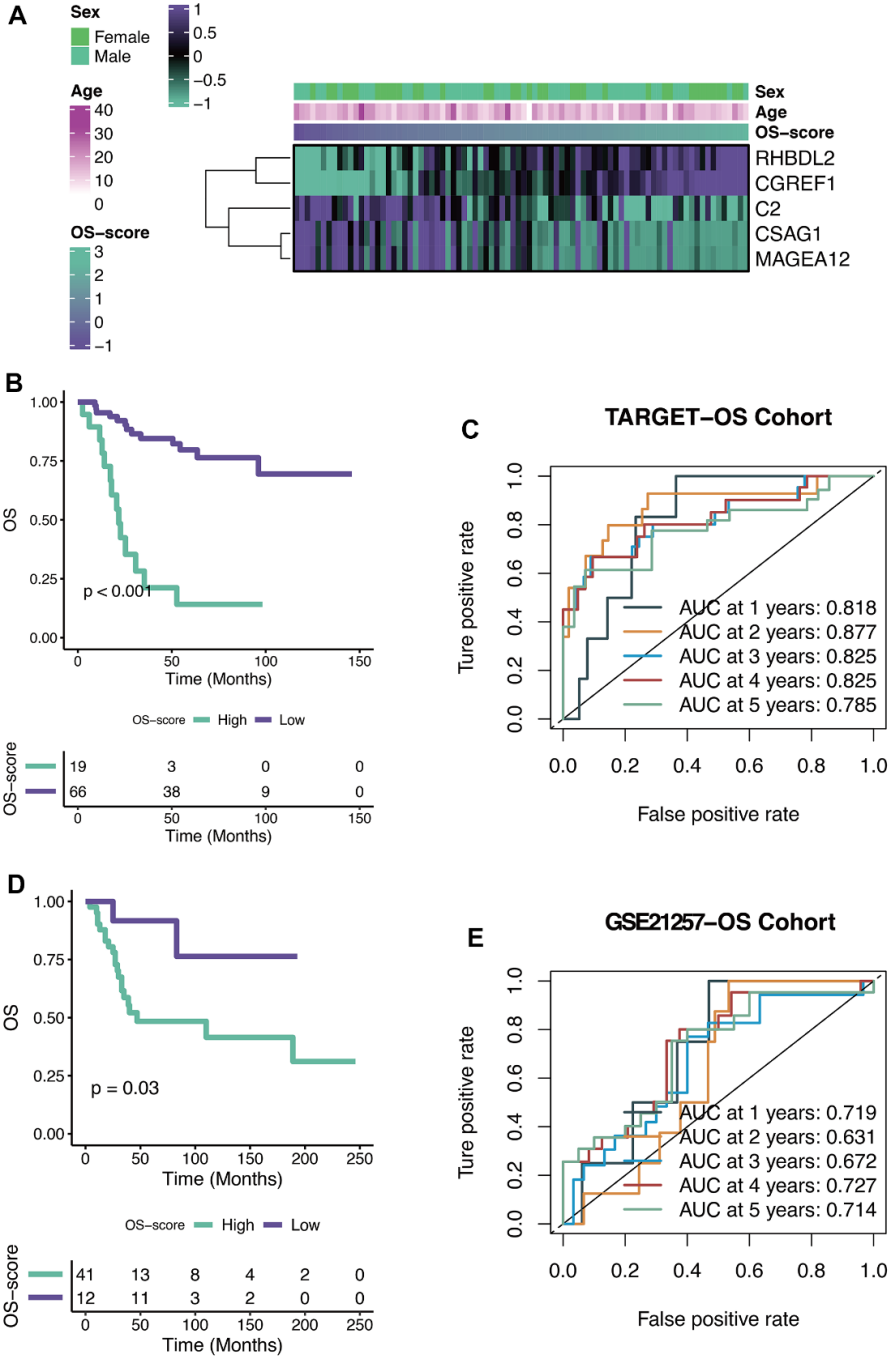
**Efficacy of OXS-signature for OS.** (**A**) Heat map showing the relationship between five genes (RHBDL2, CGREF1, C2, CSAG1, and MAGEA12) in the OXS-signature and OS-scores distribution and its clinical characteristics; (**B**) Kaplan-Meier survival curve showing survival probability of high-OS-score or low-OS-score subgroups for TARGET-OS cohort, with the green curve representing the group with higher OS-scores, and the purple curve representing the group with lower OS-scores; (**C**) The 1-year, 2-year, 3-year, 4-year, and 5-year survival ROC curves predicted by the OXS-signature for TARGET-OS cohort, with curves in different colors referring to the AUC for different years; (**D**) Kaplan-Meier survival curve showing survival probability of high-OS-score or low-OS-score subgroups for GSE21257-OS cohort, with the green curve indicating the group with higher OS-scores, and the purple curve indicating the group with lower OS-scores; (**E**) The 1-year, 2-year, 3-year, 4-year, and 5-year survival ROC curves predicted by the OXS-signature for GSE21257-OS cohort, with curves in different colors representing the AUC for different years.

The OS-scores of TARGET-OS patients were compared among the expression levels of five genes contained in the OXS-signature (Sex, Age). From the heatmap (Figure 4A), it could be found that the high expression levels of RHBDL2 and CGREF1 were associated with higher OS-scores, on the contrary, the high expression levels of C2, CSAG1 and MAGEA12 were relevant to lower OS-scores. There was no significant correlation between the two clinical factors (Sex and Age) and OS-scores. The optimal cutoff of OS-scores was set as the threshold value to distinguish TARGET-OS patients into two risk groups. As shown in the Kaplan-Meier curves (Figure 4B), significant differences in outcomes were observed between the two subgroups (P<0.001): better clinical outcomes in patients with lower OS-scores while worse clinical outcomes in patients with higher OS-scores. Therefore, OS-score might serve as a malignancy factor for OS. The 1-year (AUC=0.818), 2-year (AUC=0.877), 3-year (AUC=0.825), 4-year (AUC=0.825), and 5-year (AUC=0.785) survival ROC curves predicted by the OXS-signature revealed that the AUCs were all larger than 0.75, indicating the efficiency of the OXS-signature in predicting prognosis for TARGET-OS (Figure 4C). To further confirm the above conclusion, an external dataset (GSE21257) was included in this study. Analogously, the survival curves displayed the significantly better clinical outcomes of patients with lower OS-scores while patients with higher OS-scores experienced worse prognosis (Figure 4D, P=0.03). In addition, the 1-year (AUC=0.719), 2-year (AUC=0.631), 3-year (AUC=0.672), 4-year (AUC=0.727), and 5-year (AUC=0.714) survival ROC curves predicted by the OXS-signature also indicated the efficiency of the OXS-signature in predicting prognosis for OS patients from GSE21257 (Figure 4E).

### Immunological characteristics of OXS-signature for TARGET-OS

**Figure 5:**
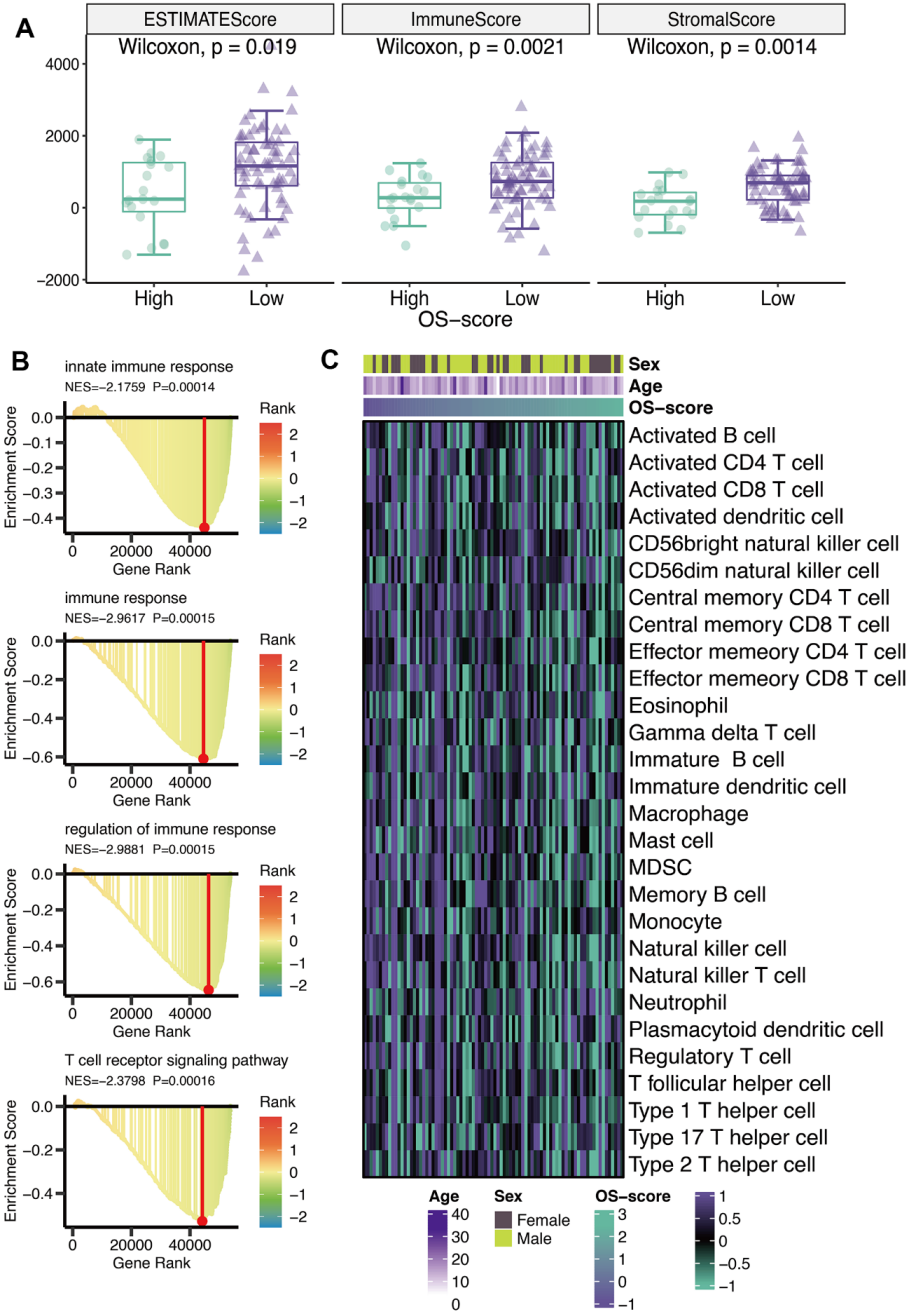
**Immunological characteristics of OXS-signature for TARGET-OS.** (**A**) The box plot comparing the differences between high- and low-OS-score on ESTIMATE score, immune score, and stromal score, with the green images representing the group with higher OS-scores, and the purple images representing the group with lower OS-scores; (**B**) GO analysis for immune-related pathways potentially regulated by OXS-signature; (**C**) The heatmap of the abundance of infiltrating immune cell populations at different OS-scores.

ESTIMATE score, immune score, and stromal score were calculated using ESTIMATE algorithm. From Figure 5A, it could be seen that all the three scores (ESTIMATE score, immune score, and stromal score) of OS patients with lower OS-scores were higher than that of OS patients with higher OS-scores (Figure 5A). GO analysis revealed that four immune-related pathways (innate immune response, immune response, regulation of immune response and T cell receptor signaling pathway) might be regulated by OS-scores (Figure 5B). To further evaluate the immunological characteristics of OXS-signature for TARGET-OS, ssGSEA analysis was carried out to represent the abundance of invasive immune cell populations with different OS-scores in the heatmap (Figure 5C).

### Immunotherapy and chemotherapy of OXS-signature for TARGET-OS

**Figure 6:**
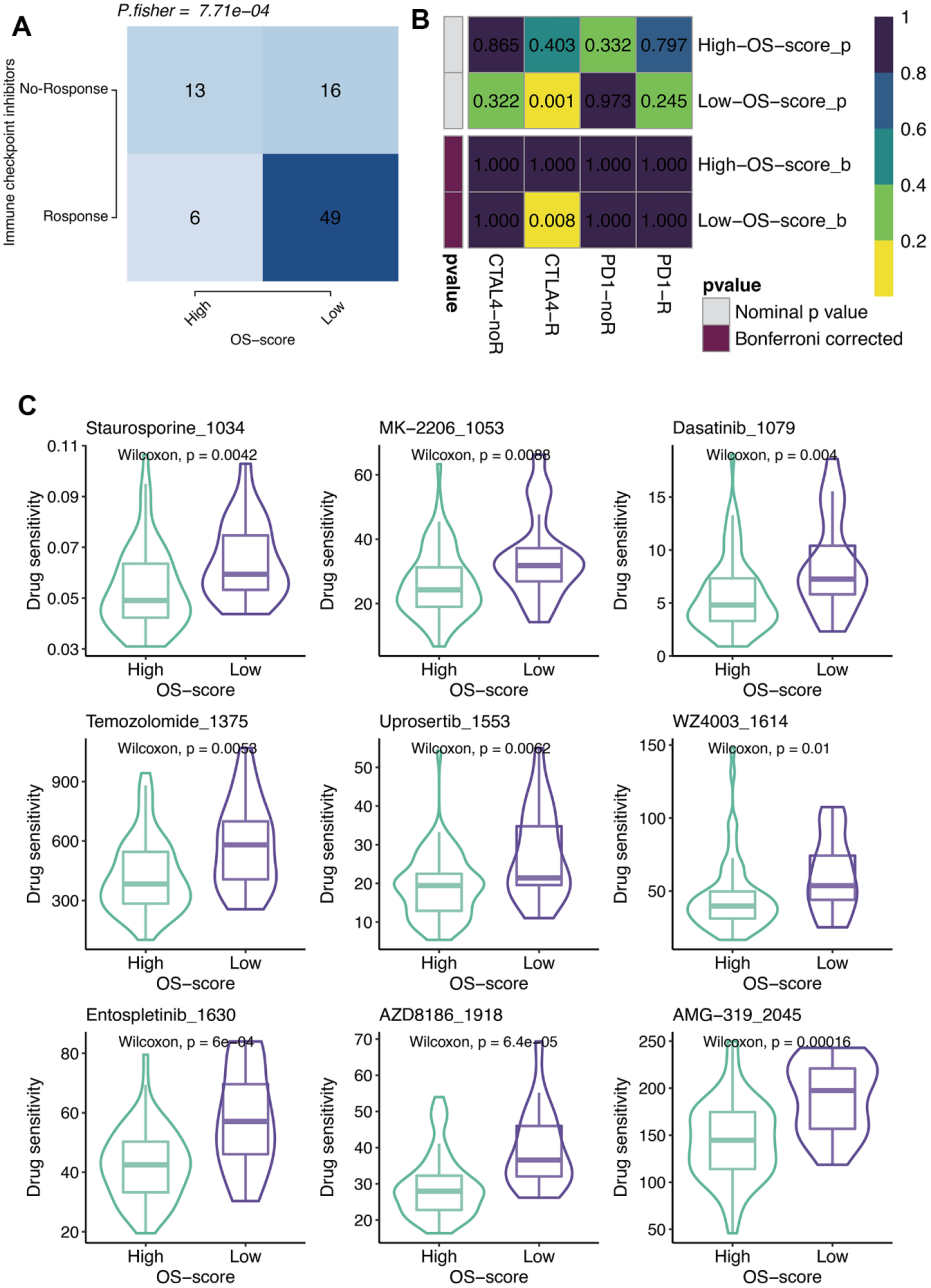
**Immunotherapy and chemotherapy of OXS-signature for TARGET-OS.** (**A**) Comparison of the ICB response rates between groups with high OS-scores or low OS-scores; (**B**) SubMap analysis for OXS-signature in TARGET-OS; (**C**) Box plots of estimated IC50 for six chemotherapeutic agents in high- or low-OS-score groups, with green images representing the group with higher OS-scores, and purple images representing the group with lower OS-scores.

According to the results of TIDE algorithm, a higher proportion of OS patients with lower OS-scores responded to immune checkpoint inhibitors, suggesting that these patients presented more sensitive to Immune Checkpoint Blockage (ICB) treatment (Figure 6A). From SubMap analysis, it could be estimated that OS patients with low OS-scores might respond to anti-CTLA4 therapy (Figure 6B). The IC50 levels of six chemotherapy drugs (Staurosporine_1034, MK-2206_1053, Dasatinib_1079, Temozolomide_1375, Uprosertib_1553, and WZ4003_1614) were compared between the two subgroups based on OS-scores. As observed, all the IC50 values of the six drugs were lower in the group with higher OS-scores than that of the group with lower OS-scores, indicating OS patients with higher OS-scores might be more sensitive to these six drugs (Figure 6C).

### RHBDL2 contributed the high expression in TARGET-OS

**Figure 7:**
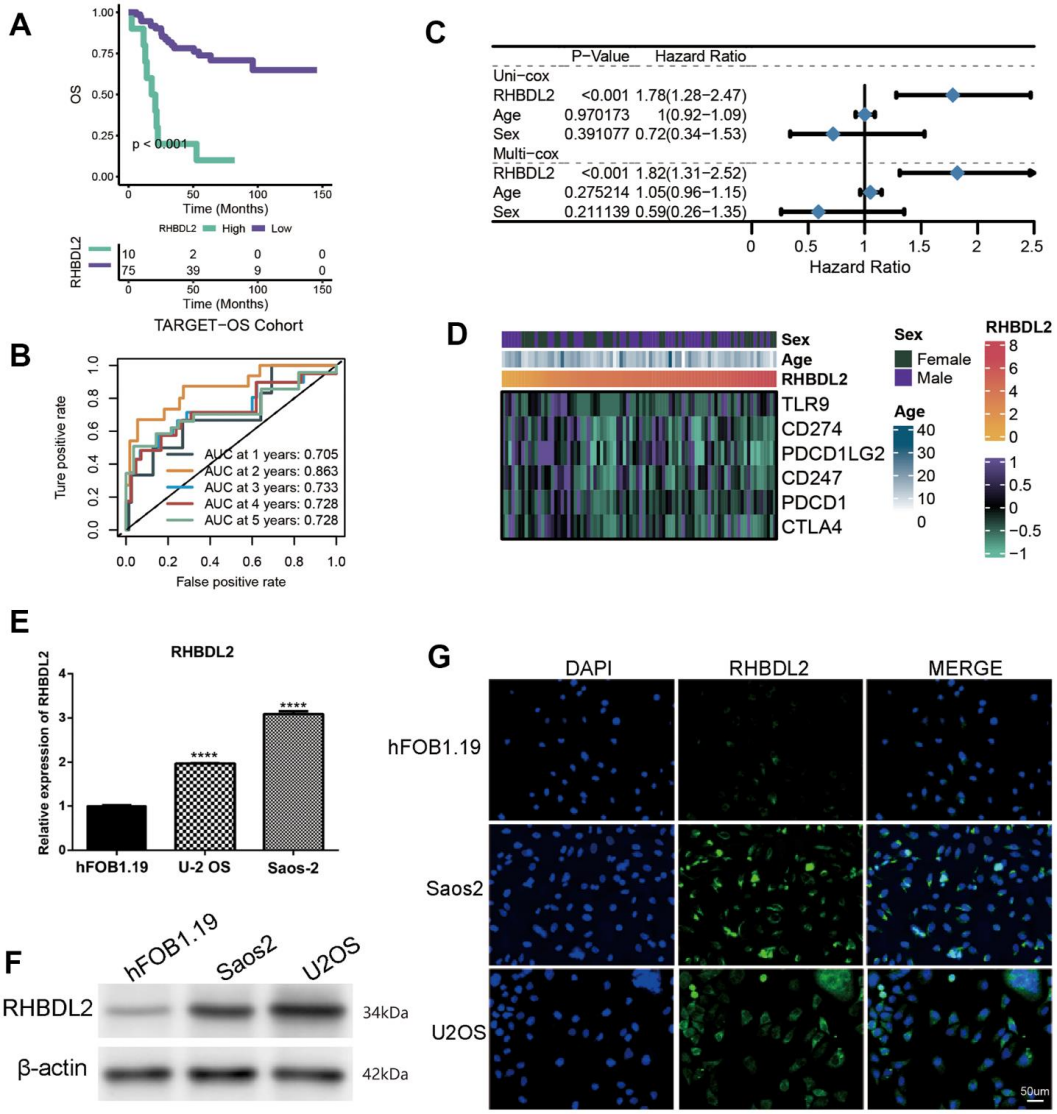
**Importance of RHBDL2 in TARGET-OS cohort.** (**A**) Kaplan-Meier survival curve showing survival probability of high- or low-expression RHBDL2; (**B**) The 1-year, 2-year, 3-year, 4-year, and 5-year survival ROC curves predicted by the expression of RHBDL2, with curves in different colors representing the AUC for different years; (**C**) The forest figure for Univariate and Multivariate Cox regression analyses of RHBDL2 and clinicopathologic features (Age, Sex); (**D**) The relationship of RHBDL2 with immune checkpoints in TARGET cohort; (**E**) Quantitative Real-time PCR. (**A**–**C**) Quantitative Real-time PCR assays using cell lines for RHBDL2; (**F**) The protein expression levels of ENO1 and CD44 in HK-2 cell control group and hyperglycemic group by Western blot; (**G**) The detection of RHBDL2 in tumor cells and normal cells by immunofluorescence.

Considering the great value of the above OXS-signature in OS prognosis and treatment, a gene, RHBDL2, in OXS-signature was randomly selected to explore its role in OS. As could be seen from the Kaplan-Meier survival curve, RHBDL2 might serve as a promoter of OS since OS patients with higher expression level of RHBDL2 appeared worse survival (Figure 7A). The AUCs for 1-year (AUC=0.705), 2-year (AUC=0.863), 3-year (AUC=0.733), 4-year (AUC=0.728), and 5-year (AUC=0.728) survival ROC curves predicted by RHBDL2 were all larger than 0.7, suggesting the efficiency of RHBDL2 in predicting prognosis for OS (Figure 7B). Moreover, Univariate and Multivariate Cox regression analyses of RHBDL2 and clinical factors confirmed that RHBDL2 was an independent prognostic factor for OS patients (Figure 7C). Additionally, it was found that RHBDL2 was significantly related to immune checkpoints (Figure 7D).

And three genes (RHBDL2, CGREF1, and C2) were randomly selected from the prognostic OXS-signature to detect their expression levels in cell lines. Compared with control cell lines (hFOB1.19), RHBDL2 (Figure 7E) and CGREF1 (Supplementary Figure 1) were markedly higher expressed in OS cell lines (U-2 OS and Saos-2), while C2 (Supplementary Figure 1) was significantly lower expressed. In addition, it was also observed that the protein of RHBDL2 was significantly increased in Osteosarcoma cell lines compared with normal cells (Figure 7F). Immunofluorescence staining was carried out on Osteosarcoma cells and normal cells. Under a confocal microscope, RHBDL2 was found to be mainly distributed in the membrane and cytoplasm, with higher expression in Osteosarcoma cells than in normal cells (Figure 7G).

## DISCUSSION

OS, a malignant tumor derived from mesenchymal tissue, is a type of sarcoma that has a high mortality rate and has become one of the killers threatening the lives of children and adolescents worldwide [1]. Due to the high heterogeneity of the tumor, there is no effective treatment at present, and surgery is still the main treatment. However, the 5-year survival rate of osteosarcoma patients treated with surgery remains low [33–35]. Currently, the relatively small number of OS specimens makes it relatively difficult to collect and analyze clinical samples. In addition, a lot of manual experimental screening inevitably costs a huge amount of manpower and material resources, and screening specific prognostic genes is even more challenging. Fortunately, with the development of cancer genomics and precise oncology, genetic information databases have sprung up. Screening of large-scale genomic datasets in large databases is conducive to narrowing the gap between cancer genomics and precise oncology and uncovering important changes in cancer etiology. Consequently, further translation of these findings into effective treatments is feasible.

With the rapid progress of molecular biology and precision oncology, sequencing technology and gene chip technology have achieved qualitative breakthroughs. At present, the methods of leveraging bioinformatics tools and database raw data to analyze tumors have been widely popularized, but the vast and precious sample information has not been deeply and comprehensively exploited. For researchers, a series of standardized and rigorous information processing methods and scientific research ideas should be gradually developed and formed, so as to make full use of them. In-depth mining of DEGs that can affect the prognosis of patients from the sample data exerts a very positive significance in promoting clinical diagnosis and treatment. In our study, based on Univariate Cox regression analysis, ten prognostic OXRGs (ACADVL, ATF4, HMOX1, GPX1, CCL2, MAPK1, MAPK14, MAP3K5, G6PD, and CAT) were identified for OS. ATF4 (activating transcription factor 4) is a stress response gene, with its expression up-regulated in response to hypoxia, amino acid deficiency, ERS, oxidative stress and some other stress factors [36, 37]. Studies have demonstrated that the ER stress response tends to occur in the case of the insufficient oxygen and nutrients supplied by tumor cells. Protein processing and folding in the ER (Endoplasmic Reticulum) require oxygen and energy. This implies that in terms of the insufficient supply of oxygen and energy, protein misfolding can lead to the occurrence of ER stress [38]. ATF4 is a major transcriptional regulatory element in cells. By binding to the promoter of C/EBP homologous protein, ATF4 is capable of inducing and activating the expression of related genes to promote correct folding of proteins and the degradation of residual proteins under endoplasmic reticulum stress (ERS) [39]. It is well acknowledged that the growth and progression of human tumors lead to the existence of tissue cells in a state of hypoxia and nutrient deprivation. Therefore, ERS response often occurs in rapidly growing and spreading cancer cells, and tumor cells also produce a series of responses to adapt to this stress state. Some studies have pointed out that compared with normal tissues, ATF4 protein is expressed at a higher level in primary human tumors, which is caused by the induction of microenvironmental factors around tumors [36, 40–42]. Previous studies have suggested that increased expression of ATF4 and MYC can induce decreased adhesion and promote cell shedding and metastasis in a variety of OS cell lines [43]. HMOX1 possesses anti-inflammatory, anti-apoptotic and anti-oxidation effects, among which the anti-oxidation effect has been extensively studied and applied [44–46]. The mechanism of anti-oxidative stress is related to Nrf2/HMOX1 signaling pathway and Nrf2/HMOX1 signaling axis plays a protective role in oxidative stress [47]. It is reported that HMOX1 is up-regulated in OS [48, 49]. Glutathione peroxidase (GPX) represents antioxidant effects [50]. The main function of GPX1 is to degrade reactive oxygen species (ROS) and protect cells from ROS. The expression products of GPX1 mainly exist in lung, liver, kidney and other tissues with high oxygen tension, and GPX1 expression is up-regulated in breast, lung and colon cancer tissues, which may be conducive to tumor progression and spread [51, 52]. However, little research has been performed on GPX1 in OS. Tumor microenvironment is closely correlated with tumor metastasis. CC chemokine ligand 2 (CCL2) is a chemokine closely related to tumor [53]. The migration of immune cells to tumors is regulated by recognizing CC chemokine ligand receptor 2 (CCR2) [53]. Metastasis-associated macrophages express high levels of CCR2 on the cell surface. The binding of CCL2 to CCR2 promotes the recruitment of macrophages to metastatic sites, thereby accelerating the dissemination and expansion of cancer cells. CCL2 can increase the metastasis and invasiveness of breast, intestinal, lung tumors, and glioblastoma multiforme [54–57]. CCL2 is associated with oxidative stress in tumors [58] and is highly expressed in OS cells [59]. ERK/MAPK pathway is one of the key signaling pathways that promote the proliferation and survival of cancer cells [60] and is widely involved in various cellular processes, such as apoptosis [61], autophagy [62], EMT [63] and cell metabolism [64]. MAPK signaling pathway exerts a great influence on the regulation of apoptosis, proliferation, invasion and metastasis of OS cells [65–68]. Glucose-6-phosphate dehydrogenase (G6PD) is the first enzyme involved in pentose phosphate pathway (PPP) and the rate-limiting enzyme of PPP. Glucose 6-phosphate produced by glycolysis is catalyzed by G6PD into PPP to generate energy needed for cellular activities [69]. Studies have manifested that G6PD expression is increased in melanoma [70], breast cancer [71], lung cancer [72, 73], liver cancer [74, 75], and colorectal cancer [76], and G6PD plays an important role in the occurrence and development of tumors. However, the remaining prognostic OXRGs have been poorly studied in tumors. The role of these OXRGs in tumors, especially OXS, still needs to be further explored.

Histopathological diagnosis has always been the gold standard in the diagnosis of OS, and is also the basis for guiding the treatment of OS [5]. However, OS is highly heterogeneous, with significant differences in treatment response and prognosis among patients with consistent histological classification and clinical staging. Individualized treatment based on molecular differences is a new direction for diagnosis and treatment of OS. Specific targeted therapy for subtypes with the same pathological origin can achieve the optimal efficacy and minimum toxicity. In recent years, with the popularization of next-generation sequencing, research of a variety of malignant tumors has been developed toward molecular targeted therapy. Given the fact that some subtypes of tumors overexpress some landmark molecules, the selection of appropriate blocking agents is beneficial for intervening and inhibiting the signal transduction pathway involved and regulated by this marker, so as to achieve the purpose of cancer suppression. However, there is no clear molecular classification of OS, which poses a major challenge for the precise treatment of OS. In our study, two OXS-clusters based on ten prognostic OXRGs were identified, providing new insights into the prognostic assessment of OS. It is worth mentioning that the identified OXS-clusters might be closely related to the immune activity of OS. Based on prognostic OXS-signature, OS patients were classified into two subtypes (patients with higher OS-scores and patients with lower OS-scores). With the improvement of OS-scores, the extent of infiltration of most immune cells appeared varying degrees of decline. Both immune cell infiltration and stromal cell scoring were lower in the high OS-score group compared to the low OS-score group. Considering the poorer prognosis of the high OS-score group and the role of immune cells in suppressing tumors, patients with higher OS-scores were believed to present a poorer tumor immune status. Tumor cells in these patients progressed more easily in the absence of immune surveillance and clearance, leading to adverse outcomes. OS patients with lower OS-scores might be more sensitive to ICB treatment. Furthermore, compared to patients with lower OS-scores, OS patients with higher OS-scores showed lower IC50 values of several chemotherapeutics (Staurosporine_1034, MK-2206_1053, Dasatinib_1079, Temozolomide_1375, Uprosertib_1553, WZ4003_1614, Entospletinib_1630, AZD8186_1918, and AMG-319 2045), suggesting that these patients revealed more sensitivity to these drugs.

Communication between OS cells and the surrounding tumor microenvironment (TME) is essential for tumor growth and subsequent metastasis. The main influencing component of TME is tumor-associated macrophages (TAM), a type of immune cells involved in inflammatory response and tissue homeostasis. There have been preliminary studies on the heterogeneity of OS tissue and immune microenvironment by single cell technique [59]. Compared with primary OS, a higher proportion of M2-type TAM has been found in lung metastases, which is associated with increased tumor invasiveness due to proinflammatory molecules. Increased invasion of TAM, especially M2 TAM, has long been connected with poor prognosis in various tumors, including OS [3]. In this study, the differences in the TME of different OS patients were explored, especially the immune microenvironment.

## Supplementary Material

Supplementary Figure 1

Supplementary Table 1

Supplementary File 1

Supplementary File 2
