# Supplementary material for: Clinical and therapeutical significances of the cluster and signature based on oxidative stress for osteosarcoma
Source: Aging (Albany NY). 2023 Dec 29;15(24):15360–81. doi: 10.18632/aging.205354 (PMC10781490; doi:10.18632/aging.205354)
Supplement: Supplementary Figure 1 [file aging-15-205354-s001.pdf]

SUPPLEMENTARY FIGURE

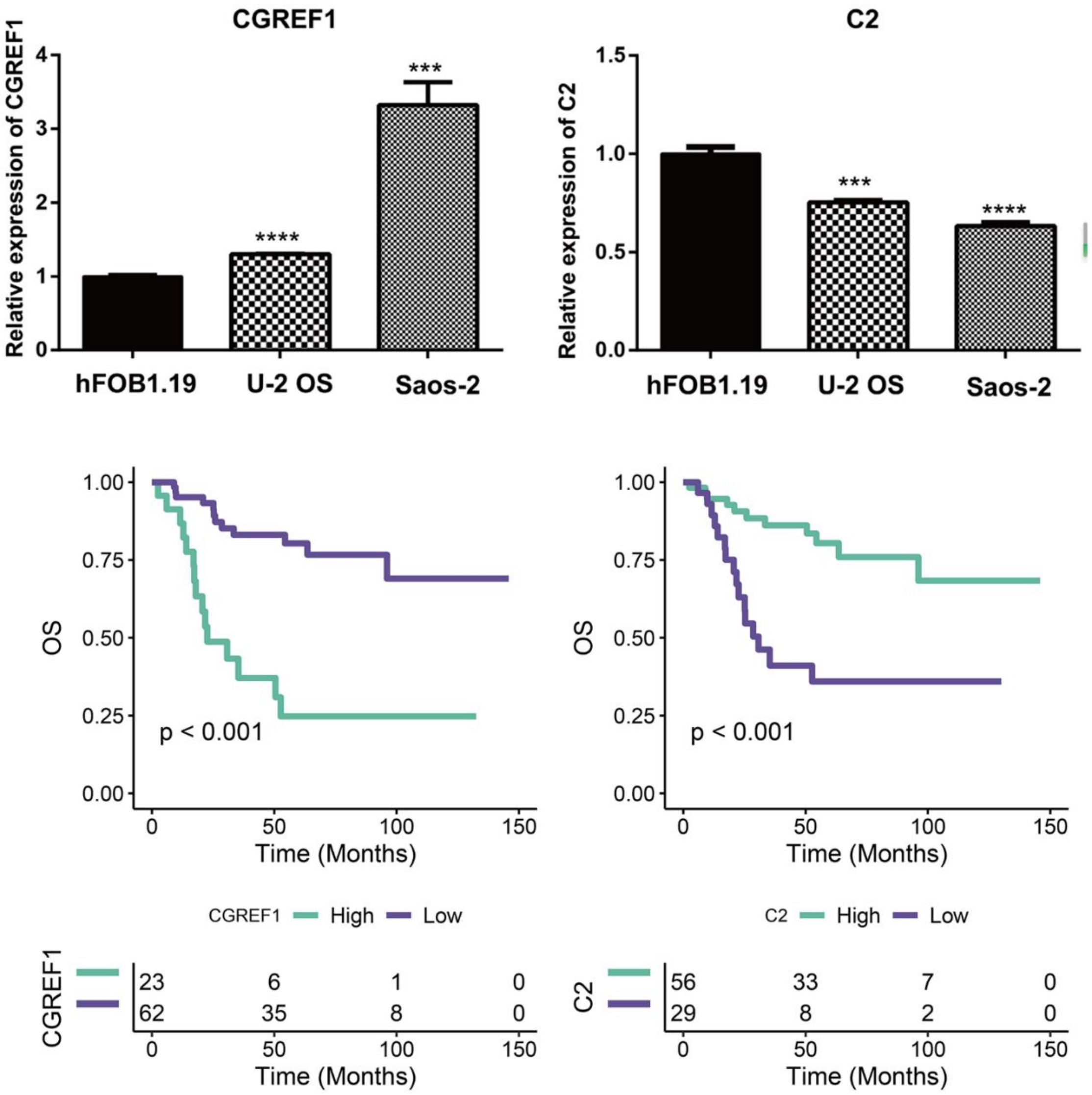

Supplementary Figure 1. Quantitative real-time PCR assays using cell lines for CGREF1 and C2.
