# Supplementary material for: Clinical and therapeutical significances of the cluster and signature based on oxidative stress for osteosarcoma
Source: Aging (Albany NY). 2023 Dec 29;15(24):15360–81. doi: 10.18632/aging.205354 (PMC10781490; doi:10.18632/aging.205354)
Supplement: Supplementary File 2 [file aging-15-205354-s004.docx]

Supplementary File 2. The code script for the bioinformatics.

FUN_surv <- function(i, surv_expr) {

Sur <- Surv(surv_expr$time, surv_expr$status)

cox <- coxph(Sur ~ surv_expr[, i], data = surv_expr)

coxSummary <- summary(cox)

gene <- names(surv_expr)[i]

HR <- coxSummary$coefficients[, "exp(coef)"]

lower <- coxSummary$conf.int[, 3]

upper <- coxSummary$conf.int[, 4]

PValue <- round(coxSummary$coefficients[, 5], 6)

res <- data.frame(gene = gene, HR = HR, lower.95 = lower, upper.95 = upper, pvalue = PValue)

return(res)

}

l <- pbapply::pblapply(3:ncol(surv_expr), FUN = FUN_surv, surv_expr = surv_expr)

Univar <- do.call(rbind, l)

Univar$gene <- Univar$gene %>% str_replace_all("_", "-")

write.csv(Univar, file = "Univar_result_OS.csv", row.names = F)

Univar <- read.csv("Univar_result_OS.csv")

# Univar$adjp <- p.adjust(Univar$pvalue, method = "BH")

Univar2 <- Univar %>% dplyr::filter(pvalue < 0.05)

dim(Univar2)

write.csv(Univar2, file = "Univar_result_OS_final.csv", row.names = F)

library(forestplot)

data <- df

head(data)

data1 <- data %>% dplyr::filter(HR > 1)

data2 <- data %>% dplyr::filter(HR < 1)

hrtable <- rbind(

c("Increase in Hazard", NA, NA, NA, NA, NA),

data1,

c("Reduce in Hazard", NA, NA, NA, NA, NA),

data2

)

# 将要在图中展示的文本

tabletext <- cbind(

c("Gene", "Increase in Hazard", data1$gene, "Reduce in Hazard", data2$gene),

c("P-value", NA, data1$pvalue, NA, data2$pvalue),

c("Hazard Ratio", NA, data1$`HR (95%CI)`, NA, data2$`HR (95%CI)`)

)

which(tabletext[, 1] == "Reduction in Hazard")

nrow(tabletext) + 1

quantile(df$lower)

quantile(df$upper)

pdf("forestplot_OS_genes.pdf", width = 10, height = 6) # 新建PDF文件，准备写入图形

forestplot(

labeltext = tabletext, # 图中的文本

mean = c(NA, as.numeric(hrtable$HR)), # HR

lower = c(NA, as.numeric(hrtable$lower)), # 95%置信区间下限

upper = c(NA, as.numeric(hrtable$upper)), # 95%置信区间上限

# title="Hazard Ratio",

graph.pos = 4, # 图在表中的列位置

graphwidth = unit(.4, "npc"), # 图在表中的宽度比例

fn.ci_norm = "fpDrawDiamondCI", # box类型选择钻石

col = fpColors(box = "steelblue", lines = "black", zero = "black"), # box颜色

# col = fpColors(box = "#1982c4", lines = "#1982c4", zero = "black"), # box颜色

boxsize = 0.7, # box大小根据样本量设置

lwd.ci = 3, ci.vertices.height = 0.125,

ci.vertices = T, # 置信区间用线宽、高、型

zero = 1, # zero线横坐标

lwd.zero = 2,

xticks = seq(0, 4, 1), # 横坐标刻度根据需要可随意设置

lwd.xaxis = 2, # X轴线宽

xlab = "Hazard Ratio",

hrzl_lines = list(

"1" = gpar(lwd = 2, col = "black"), # 第二行顶部加黑实线

"2" = gpar(lwd = 1, col = "grey50", lty = 2), # 第二行顶部加灰色虚线

"5" = gpar(lwd = 1, col = "grey50", lty = 2), # 第九行顶部加灰色虚线

"14" = gpar(lwd = 2, col = "black")

), # 最后一行底部加黑线，""中数字为nrow(tabletext) + 1

lineheight = unit(0.8, "cm"), # 固定行高

txt_gp = fpTxtGp(

label = gpar(cex = 1), # 各种字体大小设置

ticks = gpar(cex = 1.25),

xlab = gpar(cex = 1.85),

title = gpar(cex = 1.5)

),

# align=c("l","c","c"),#每列文字的对齐方式，偶尔会用到

clip = c(0, 2),

colgap = unit(0.15, "cm"), # 列间隙

mar = unit(rep(1.25, times = 4), "cm"), # 图形页边距

new_page = F # 是否新页

)

dev.off()

seed <- 2020

maxK <- 10 # maximum number of clusters to try

results <- ConsensusClusterPlus(d, tmyPal = c("#64BFA5", "#67529C"),

maxK = maxK, reps = 1000, pItem = 0.8, pFeature = 1, title = "cluster_TARGET",

innerLinkage = "complete", seed = seed, plot = "pdf", clusterAlg = "kmdist", corUse = "complete.obs", writeTable = T

) # Note that we implement consensus clustering with innerLinkage="complete".

# save(results, file = "ConsensusCluster_ssGSEA_TARGET.RData")

# We advise against using innerLinkage="average" which is the default value in this package as average linkage is not robust to outliers.

############## PAC implementation ##############

Kvec <- 2:maxK

x1 <- 0.1

x2 <- 0.9 # threshold defining the intermediate sub-interval

PAC <- rep(NA, length(Kvec))

names(PAC) <- paste("K=", Kvec, sep = "") # from 2 to maxK

for (i in Kvec) {

M <- results[[i]]$consensusMatrix

Fn <- ecdf(M[lower.tri(M)])

PAC[i - 1] <- Fn(x2) - Fn(x1)

} # end for i# The optimal K

optK <- Kvec[which.min(PAC)]

optK

sel1 <- read.csv("clustergsea_result_KEGG_cluster_tumor.csv") %>% dplyr::slice(1:3)

sel2 <- read.csv("clustergsea_result_KEGG_cluster_imm.csv") %>% dplyr::slice(1:3)

sel <- rbind(sel1, sel2)

clustergsea <- clustergsea[clustergsea$ID %in% sel$ID, asis = T]

res <- clustergsea@result

source("GSEA_KEGG_plot.R")

FUN <- function(i) {

tmp <- res[which(res$ID == i), ]

lab <- str_c("NES=", round(tmp$NES, 4), " P=", format(tmp$pvalue, digits = 2, scientific = F))

p <- GSEA_KEGG_plot(object = clustergsea, geneSetID = i, mRNA_si = mRNA_si, text = F, subtitle = lab)

return(p)

}

l <- lapply(sel$ID, FUN)

p <- cowplot::plot_grid(plotlist = l, nrow = 2)

ggsave(plot = p, filename = "GSEAplot_cluster.pdf", width = 12, height = 6)

x <- coef(fit)

tmp <- as.data.frame(as.matrix(x))

tmp$coef <- row.names(tmp)

tmp <- reshape::melt(tmp, id = "coef")

tmp$variable <- as.numeric(gsub("s", "", tmp$variable))

tmp$coef <- gsub("_", "-", tmp$coef)

tmp$lambda <- fit$lambda[tmp$variable + 1] # extract the lambda values

tmp$norm <- apply(abs(x[-1, ]), 2, sum)[tmp$variable + 1] # compute L1 norm

library(ggsci)

mycol <- c("#223D6C","#D20A13","#FFD121","#088247","#11AA4D","#58CDD9","#7A142C","#5D90BA","#029149","#431A3D","#91612D","#6E568C","#E0367A","#D8D155","#64495D","#7CC767")

p1 <- ggplot(tmp, aes(log(lambda), value, color = coef)) +

geom_vline(xintercept = log(cv.fit$lambda.min), size = 0.8, color = "grey60", alpha = 0.8, linetype = 2) +

geom_line(size = 1) +

xlab("Lambda (log scale)") +

# xlab("L1 norm")+

ylab("Coefficients") +

theme_pubr() +

scale_color_manual(values = mycol) +

scale_x_continuous(expand = c(0.05, 0.05)) +

scale_y_continuous(expand = c(0.01, 0.01)) +

theme(

panel.grid = element_blank(),

axis.title = element_text(size = 15, color = "black"),

axis.text = element_text(size = 12, color = "black"),

legend.title = element_blank(),

legend.text = element_text(size = 12, color = "black"),

legend.position = "right"

) +

# annotate('text',x = -3.3,y=0.26,label='Optimal Lambda = 0.012',color='black')+

guides(col = guide_legend(ncol = 1))

# ggsave("lasso_A_fs.pdf", width = 6, height = 4)

p2 <- ggplot(xx, aes(ll, cvm, color = NZERO)) +

geom_errorbar(aes(x = ll, ymin = cvlo, ymax = cvup), width = 0.05, size = 1) +

geom_vline(xintercept = xx$ll[which.min(xx$cvm)], size = 0.8, color = "grey60", alpha = 0.8, linetype = 2) +

geom_point(size = 2) +

xlab("Log Lambda") +

ylab("Partial Likelihood Deviance") +

theme_pubr() +

scale_color_manual(values = mycol) +

scale_x_continuous(expand = c(0.02, 0.02)) +

scale_y_continuous(expand = c(0.02, 0.02)) +

theme(

panel.grid = element_blank(),

axis.title = element_text(size = 15, color = "black"),

axis.text = element_text(size = 12, color = "black"),

legend.title = element_blank(),

legend.text = element_text(size = 12, color = "black"),

legend.position = "right"

) +

# annotate('text',x = -5.3,y=12.4,label='Optimal Lambda = 0.008',color='black')+

guides(col = guide_legend(ncol = 1))

# ggsave("lasso_B_fs.pdf", width = 6, height = 4)

cowplot::plot_grid(p1, p2, nrow =1, align = 'hv')

ggsave("Lasso_plot.pdf", width = 12, height = 3.5)

adjp <- p.adjust(p, 'bonferroni')

tmp <- matrix(c(

p, adjp

), # Bonferroni校正p值

nrow = 4, byrow = T, dimnames = list(c("High-OS-score_p", "Low-OS-score_p", "High-OS-score_b", "Low-OS-score_b"), c("CTAL4-noR", "CTLA4-R", "PD1-noR", "PD1-R"))

)

library(pheatmap)

pheatmap(tmp,

cellwidth = 30, cellheight = 30,

cluster_rows = F, cluster_cols = F,

color = heatmap.YlGnPe[5:1], number_format = "%.3f",

gaps_row = 2, display_numbers = T, number_color = "black", fontsize_number = 10,

annotation_row = data.frame(pvalue = c("Nominal p value", "Nominal p value", "Bonferroni corrected", "Bonferroni corrected"), row.names = rownames(tmp)),

annotation_colors = list(pvalue=c("Nominal p value"=lightgrey,"Bonferroni corrected"=cherry)),

filename = "heatmap_submap.pdf"

)
